# Incidence, Risk Factors, Treatment and Prognosis of Popliteal Artery Embolization in the Superficial Femoral Artery Interventions

**DOI:** 10.1371/journal.pone.0107717

**Published:** 2014-09-19

**Authors:** Weiwei Wu, Surong Hua, Yongjun Li, Wei Ye, Bao Liu, Yuehong Zheng, Xiaojun Song, Changwei Liu

**Affiliations:** 1 Department of Vascular Surgery, Peking Union Medical College Hospital, Chinese Academy of Medical Science, Beijing, China; 2 Department of Vascular Surgery, Beijing Tsinghua Changgung Hospital, Beijing Tsinghua University, Beijing, China; Hokkaido University, Japan

## Abstract

**Objective:**

Percutaneous transluminal angioplasty and stenting (PTA + stent) has gained acceptance as a primary treatment modality for the superficial femoral artery (SFA) diseases. Popliteal artery embolization (PAE) is a severe complication in SFA interventions. The purpose of this study was to evaluate the incidence, risk factors, treatment and prognosis of PAE in primary SFA PTA + stent.

**Methods:**

Chronic SFA arteriosclerosis cases that underwent primary PTA + stent were reviewed from a retrospectively maintained database. Runoff vessels were evaluated in all cases before and after the interventions for PAE detection. The primary patency, secondary patency and limb salvage rates were calculated using Kaplan-Meier analysis and compared using log-rank analysis. Cox multivariate regression was performed to evaluate predictors of patency and limb salvage rates.

**Results:**

There were 436 lesions treated in 388 patients with 10 PAE events (2.3%) in total. PAE rate was significantly higher in Transatlantic Inter-Society Consensus (TASC) C/D group compared with TASC A/B group (OR = 8.91, *P* = .002), in chronic total occlusion (CTO) lesions compared with stenotic lesions (*P*<.0001), and in group with history of cerebral ischemic stroke (OR = 6.11, *P* = .007). PAE rates were not significantly affected by age, sex, smoking, hypertension, diabetes, hyperlipidemia and runoff status. The binary logistic regression showed that only the TASC C/D was an independent predictor of PAE (*P* = .031). The 12-month and 24-month primary patency, secondary patency and limb salvage rates in PAE group showed no significant differences comparing with non-PAE group.

**Conclusions:**

PAE is a rare event in primary SFA PTA + stent. TASC C/D lesion, CTO and cerebral ischemic stroke history are risk factors for PAE. PAE is typically reversible by comprehensive techniques. If the popliteal flow is restored in time, PAE has no significant effect on long-term patency and limb salvage rates.

## Introduction

Distal embolization (DE) of thromboembolic material generated during lower extremity endovascular intervention is a known complication following potential severe ischemic consequences [1]. The reported incidence of DE, detected angiographically or clinically, ranges from 1% to 5% [2]. DE may necessitate additional intervention, including thrombectomy or thrombolysis, resulting in longer procedure time, more contrast used, and greater radiation exposure [3]. The concern for DE in lower extremity interventions has led to a debate [4]. Some recommend the use of a variety of embolic protection devices (EPDs) [5], [6], [7], while other evidence suggested that EPDs may be unnecessary [2], [8]. Clinical data have shown that the application of EPDs in lower extremity is generally safe [4]. More trial data may be necessary to find the balance between the increase in cost, complexity, risks and the potential benefit [9]. DE rate can differ a lot in different lesion types and treatment methods. It was reported that reintervention may have a higher rate of DE, and the use of newer atherectomy devices may be more emboligenic than angioplasty with or without stenting [2]. However, it is still not quite sure to tell which type of lesion or treatment methods could benefit from EPDs. More retrospective or prospective studies need to be done to identify the incidence and prognosis of DE in each subgroup.

The superficial femoral artery (SFA) is one of the most common affected target vessels in the atherosclerotic occlusive disease of the lower extremities. For most doctors, endovascular therapy, especially percutaneous transluminal angioplasty and stenting (PTA + stent), has become the primary choice for SFA occlusion. However, DE remains a concern in SFA interventions. Popliteal artery embolization (PAE) is the most severe type of DE. The acute occlusion before popliteal trifurcation in SFA interventions may cause sudden pain, worsening ischemia or later limb loss, which will be a nightmare for both patient and operator.

It is reported that DE has no effects on patency rates and limb salvage for all kinds of lower extremity interventions [2]. But this previous research did not separate PAE from other runoff vessel embolization, nor PTA + stent from other interventions. So far there have been no literatures clearly demonstrating the rate, treatment and prognosis of PAE in primary SFA PTA + stent. This study was aim to evaluate the incidence, risk factors, treatment and prognosis of PAE in chronic SFA arteriosclerosis cases underwent primary PTA + stent.

## Methods

### Patient Selection

All patients treated percutaneously for chronic lower extremity ischemia with atherosclerotic occlusive disease in a single center were identified in a retrospectively maintained database. All SFA cases that underwent primary PTA + stent from November 2008 to December 2012 were reviewed. Indications for intervention were peripheral arterial disease greater than Rutherford's category 2, including moderate to severe claudication or critical limb ischemia, defined as rest pain, tissue loss, or non-healing ulceration. All patients suffered from lower extremity ischemia for at least 3 months, and all procedures were performed by 4 experienced vascular surgeons. The medication included: routine antiplatelet therapy (at least one week of aspirin 100 mg daily prior and indefinitely after the procedure, and clopidogrel 75 mg daily for three months after the procedure) and intensive lipid-lowering therapy (mostly atorvastatin). Patients with acute or subacute limb ischemia less than 3 months were excluded. The study protocol was reviewed and approved by the Ethics Committee of Peking Union Medical College Hospital. Written informed consents for both the procedure and the use in anonymous observational research were collected from all patients.

### Treatments and Technique

PTA + stent was carried out in all cases. Most procedures were performed under local anesthesia. A crossover approach from the contralateral side was established and a long sheath was placed for the proximal SFA lesions, while an antegrade transfemoral access was selected for the ipsilateral middle or distal SFA lesions. Retrograde or transbrachial approaches were not used in this group. Anticoagulation included 80–100 unit/kg of unfractionated heparin given intravenously at the beginning of the procedure and another 500–1000 unit/h during the procedure to maintain an activated clotting time between 250 and 300 seconds (maximum total dose of heparin 10,000 units). Angiography of the total SFA and distal runoff was performed in all cases. The 0.035 inch hydrophilic guide wires were used to cross the stenosis lesions, and appropriated sized self-expanding bared stents (often 6 or 7 mm in diameter) were deployed overlapping the lesions. The 0.018 inch or 0.014 inch guide wires supported by a noncompliant balloon (often 2.5 or 3.0 mm in diameter) were usually chosen to cross the occluded lesions with either an intraluminal or subintimal technique. After the wires got back into the distal true lumen, the supported balloons were inflated to its nominal pressure to pre-dilate the occluded lesions, in order to facilitate the subsequent delivery of the self-expanding bare stents. Post-dilation was then performed with noncompliant balloons (often 1 mm less than the stent in diameter) within the stents. Completion angiography with evaluation of the distal runoff was performed after all interventions.

In case of PAE, the salvage intraluminal techniques including aspiration with guiding catheters, local thrombolysis with urokinase, and/or angioplasty with small sized noncompliant balloons would be used at once. If all of the above did not open the popliteal artery occlusion, popliteal artery embolectomy through a below-knee medial longitudinal incision would be performed as quickly as possible under general anesthesia.

### Data Collection and Follow-up

Operative reports and angiograms were examined by two separate reviewers to determine lesion type and Transatlantic Inter-Society Consensus (TASC) II classification [10]. The lesion with angiography proved blood flow interruption at SFA segment was defined as chronic total occlusion (CTO) lesion, otherwise was defined as stenotic lesion. Runoff vessel evaluation (including the popliteal, anterior tibial, posterior tibial and peroneal artery) was performed in all cases before and after intervention for PAE detection. PAE was defined as new onset of angiographic contrast filling defect in the popliteal artery with all below-knee runoff vessels blocked at any time during the procedure, other than vasospasm and dissection. Patients were followed up at 1, 3, 6, and 12 months after the interventions, and annually thereafter. Physical examination (ankle-brachial index and pulse examination) and duplex ultrasonography (DUS) were performed on each follow-up visit. Follow-up angiography was indicated when the findings on DUS suggested restenosis over 50 percent. Loss of primary or secondary patency was defined as the presence of over 50% stenosis during angiography after the primary or secondary intervention. Loss of limb salvage was defined as any amputation above the level of the ankle joint.

### Statistical Analysis

Chi-square analysis or Fisher's exact test were used to assess significance based on *P*<.05 for categorical data. Odds ratio (OR) and 95% confidence intervals (CI) were also calculated when *P*<.05. The primary patency, secondary patency and limb salvage rates were calculated using Kaplan-Meier analysis and compared using log-rank analysis. Cox multivariate regression was performed to evaluate whether PAE and other factors were predictor of decreased patency and limb salvage rates. All analysis was done with SPSS 19.0 software (SPSS Inc, Chicago).

## Results

There were 436 lesions treated in 388 patients. Demographic data and comorbidities are outlined in Table 1. Two hundred and ninety two patients (75.3%) were men and 96 patients (24.7%) were women. The average age was 68.9±8.5 years (range from 44 to 87 years). Lesion types and characteristics are listed in Table 2. One hundred and ninety one lesions (43.8%) were in Transatlantic Inter-Society Consensus (TASC) II A group, while 105 (24.1%), 51 (11.7%) and 89 (20.4%) lesions were in TASC B, C and D group, respectively.

**Table 1:** Patient demographics and comorbidities.

Variable	
Total patients	388 (100.0%)
Age (years)	68.9±8.5
Male	292 (75.3%)
Smokers (current and former)	184 (47.4%)
Hypertension	281 (72.4%)
Diabetes mellitus	208 (53.6%)
Hyperlipidemia	153 (39.4%)
Coronary artery disease	108 (27.8%)
Cerebral ischemic stroke	81 (20.9%)
Chronic renal insufficiency	8 (2.1%)
Other peripheral artery diseases	70 (18.0%)
Rutherford's category	
2	35 (9.0%)
3	207 (53.4%)
4	72 (18.6%)
5	58 (14.9%)
6	16 (4.1%)

Continuous data are presented as means ± standard deviation; categorical data are given as counts (percentages).

**Table 2:** Lesion types and characteristics.

Variable	
Total lesions	436
Lesion type	
Stenosis	278 (63.8)
Chronic total occlusion	158 (36.2)
TASC II classification	
A	191 (43.8)
B	105 (24.1)
C	51 (11.7)
D	89 (20.4)
A/B (low TASC grade)	296 (67.9)
C/D (high TASC grade)	140 (32.1)
Preoperative runoff vessels	1.82±0.81
Preoperative runoff	
0	9 (2.1)
1	160 (36.7)
2	166 (38.1)
3	101 (23.2)
0/1 (insufficient runoff)	169 (38.8)
2/3 (sufficient runoff)	267 (61.2)

Continuous data are presented as means ± standard deviation; categorical data are given as counts (percentages).

TASC, Transatlantic Inter-Society Consensus.

There were 15 DE events (3.4%) in the popliteal, tibial or peroneal artery, ten of which were PAE. The other 5 DE events located in the tibial or peroneal artery with the popliteal artery and at least one other runoff vessel patent showed no symptoms, thus, no specific treatments were carried out. Table 3 summarizes and compares the rates of PAE in different groups. There were 10 PAE events (2.3%) in total, of which 2 happened in TASC B group, 2 in C group and 6 in D group. The PAE rate was significantly higher in TASC C/D group compared with TASC A/B group (OR = 8.91, 95% CI: 1.87–42.53, *P* = .002), in CTO lesions compared with stenotic lesions (*P*<.0001), and in group with history of cerebral ischemic stroke (OR = 6.11, 95% CI: 1.69–22.13, *P* = .007). The mean age was 67.0±4.6 years in PAE group compared with 68.6±8.6 years in non-PAE group (*P* = .553). PAE rates were not significantly affected by age, sex, smoking, hypertension, diabetes, hyperlipidemia and runoff status. The binary logistic regression showed that only the TASC C/D was an independent predictor of PAE (*P* = .031), while others were not. We noticed that all 10 PAE events happened in male patients and CTO lesions. Within the CTO group, PAE rates in the intraluminal (5.38%, 5/93) and subintimal subgroup (7.69%, 5/65) were not significantly different (*P* = .915).

**Table 3:** PAE rates in different subgroups.

Subgroup	Lesions	PAE	*P* value
	Total No.	No. (%)	
Male	320	10 (3.1%)	.069
Female	116	0 (0%)	
Smokers (current and former)	210	4 (1.9%)	.753
Non-smokers	226	6 (2.7%)	
Hypertension	312	6 (1.9%)	.480
Without hypertension	124	4 (3.2%)	
Diabetes mellitus	235	4 (1.7%)	.524
Without diabetes mellitus	201	6 (3.0%)	
Hyperlipidemia	175	4 (2.3%)	1.000
Without hyperlipidemia	261	6 (2.3%)	
Coronary artery disease	109	4 (3.7%)	.276
Without coronary artery disease	327	6 (1.8%)	
Cerebral ischemic stroke	90	6 (6.7%)	**.007** a
Without cerebral ischemic stroke	346	4 (1.6%)	
Chronic renal insufficiency	8	0 (0%)	1.000
Without chronic renal insufficiency	428	10 (2.3%)	
Lesion type			
Chronic total occlusion	158	10 (6.3%)	**<.0001**
Stenosis	278	0 (0%)	
TASC II classification			
C/D (high TASC grade)	140	8 (5.7%)	**.002** b
A/B (low TASC grade)	296	2 (0.7%)	
Preoperative runoff			
2/3 (sufficient runoff)	267	8 (3.0%)	.329
0/1 (insufficient runoff)	169	2 (1.2%)	

Categorical data are given as counts (percentages).

PAE, Popliteal artery embolization; TASC, Transatlantic Inter-Society Consensus.

*P* values of <.05 are in bold.

a Odds ratio (OR) = 6.11, 95% confidence interval (CI): 1.69–22.13.

b OR = 8.91, 95% CI: 1.87–42.53.

**Figure 1:**
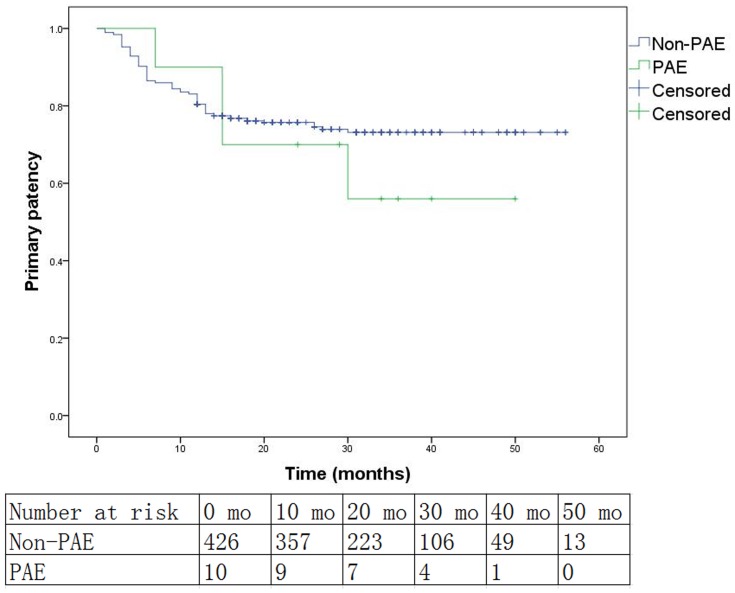
Kaplan-Meier curves for primary patency between the popliteal artery embolization (PAE) group and the non-PAE group (Log rank test: *P* = .475).

**Figure 2:**
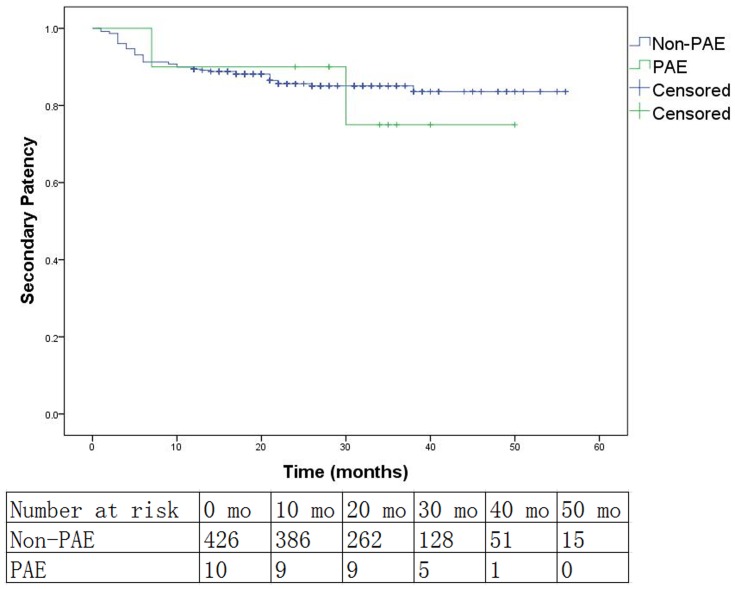
Kaplan-Meier curves for secondary patency between the popliteal artery embolization (PAE) group and the non-PAE group (Log rank test: *P* = .736).

**Figure 3:**
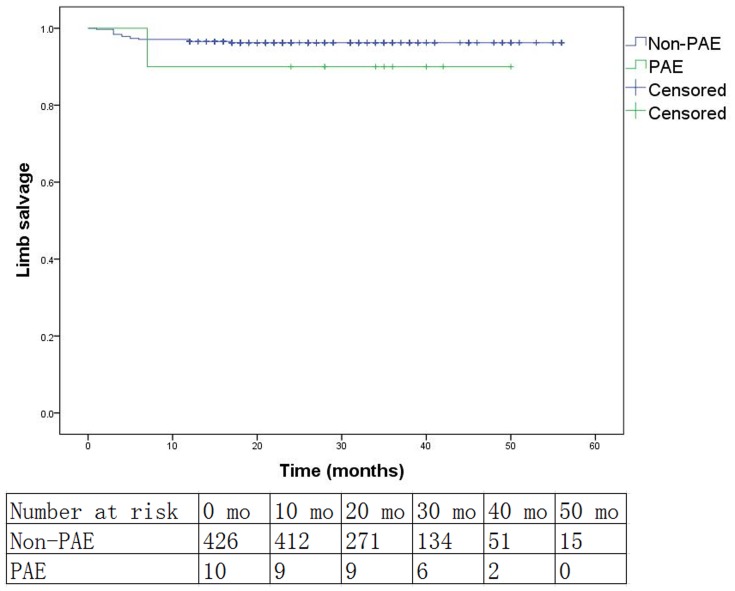
Kaplan-Meier curves for limb salvage between the popliteal artery embolization (PAE) group and the non-PAE group (Log rank test: *P* = .298).

Patency of the popliteal artery and at least one runoff vessel was restored at the completion of the procedure in all cases of PAE. Aspiration and local thrombolysis were not working in any case. Angioplasty with noncompliant balloons (3.0 or 3.5 mm in diameter) had opened the PAE and restored the flow of at least one below-knee runoff vessel in 6 cases. Embolectomy was performed immediately in the rest 4 cases that were not salvaged by the intraluminal techniques. Pathology exams showed that all emboli were atheromatous plaque rather than thrombus. Kaplan-Meier curves and log-rank test analysis results of the primary patency, secondary patency and limb salvage rates in the PAE and non-PAE group are shown in Figs. 1, 2 and 3. The median follow-up time of the PAE group is 29.5 months (7–50 months), while that of the non-PAE group is 20 months (1–56 months). The dropout percentage of follow-up is 7.8% (34/436). The overall primary patency (*P* = .475), secondary patency (*P* = .736) and limb salvage rates (*P* = .298) between the PAE and non-PAE group show no significant differences. At 12 months, the two groups (PAE vs. non-PAE) were equivalent in terms of primary patency (PAE: 80.0%, non-PAE: 80.1%, *P* = 1.000), secondary patency (PAE: 90%, non-PAE: 89.6%, *P* = 1.000), and limb salvage (PAE: 90%, non-PAE: 96.7%, *P* = .298). There were also no significant differences between the two groups at 24 months or 36 months in primary patency, secondary patency, or limb salvage (*P*>.05, Table 4). Cox multivariate regression model showed that PAE was not an independent predictor of risk in primary patency, secondary patency or limb salvage (*P*>.05, Table 5). Diabetes mellitus and hyperlipidemia were independent predictors of risk for primary patency, while hyperlipidemia and CTO were independent predictors for poor secondary patency. However, we found that hypertension was associated with decreased risk in primary patency.

**Table 4:** Comparison of patency and limb salvage rates.

Patency rate	12 months	24 months	36 months
	value ± SE	value ± SE	value ± SE
Primary patency			
Non-PAE	80.1±1.9%	75.4±2.1%	72.5±2.4%
PAE	80.0±12.6%	70.0±14.5%	56.0±17.1%
*P* value	1.000	.714	.474
Secondary patency			
Non-PAE	89.6±1.5%	85.9±1.8%	84.8±1.9%
PAE	90.0±9.5%	90.0±9.5%	75.0±15.8%
*P* value	1.000	1.000	.656
Limb salvage			
Non-PAE	96.7±0.9%	96.4±0.9%	96.4±0.9%
PAE	90.0±9.5%	90.0±9.5%	90.0±9.5%
*P* value	.298	.315	.315

PAE, Popliteal artery embolization; SE, Standard error.

**Table 5:** Cox multivariate analysis of risk factors.

Variable	HR (95% CI)	*P* value
Primary patency		
PAE	1.60 (0.54–4.79)	.401
Diabetes mellitus	2.14 (1.39–3.28)	.001
Hypertension	0.63 (0.41–0.97)	.035
Hyperlipidemia	1.67 (1.12–2.50)	.013
Secondary patency		
PAE	0.92 (0.20–4.22)	.912
Hyperlipidemia	2.51 (1.43–4.40)	.001
Chronic total occlusion	1.93 (1.07–3.47)	.028
Limb salvage		
PAE	4.35 (0.36–52.16)	.246

CI, Confidence interval; HR, hazard ratio; PAE, Popliteal artery embolization.

## Discussion

Contemporary studies have demonstrated a higher rate of DE in the filter of EPDs (20–58%) [3], [7], [11], or detected by Doppler ultrasound (100%) [8], [12], than angiographically (1%–5%) [1], [2]. The reported incidence of limb-threatening DE during routine lower extremity intervention is 1–2% [1], [13]. Most of the limb-threatening DE are PAE according to our experience. The incidence of PAE was seldom discussed in literature, and our data suggests that PAE rate (2.3%) is similar to limb-threatening DE rate. Published research reported that DE rate was significantly higher in CTO lesions compared with stenotic lesions, and in TASC C/D group compared with TASC A/B group [2]. We found the results were consistent with PAE. TASC C/D lesion, CTO lesion and history of cerebral ischemic stroke are risk factors for PAE. Aspiration and local thrombolysis were not working in all PAE cases, and all emboli retrieved from embolectomy were atheromatous plaques, suggesting that the embolic debris was mainly made of atheromatous plaque, not thrombus. The possible mechanism for PAE may be that debris is more likely to drop or release from unstable plaque in TASC C/D or CTO lesions during the intervention, in course of which the guide wire or other devices are often used to pass the occlusion by force. Once the occluded lesion is open, the debris will be flushed to the distal vessels by chance. There is little clinical evidence about cerebral ischemic stroke increases DE rate in peripheral intervention as far as we know. But basic research showed that several genes were associated with altered macrophage activity or endothelial function, resulting in increased stroke incidence and unstable plaque in cerebrovascular diseases [14], [15]. It is reasonable to presume that in patients with history of cerebral ischemic stroke in this study might be inclined to form unstable plaque in the SFA lesions, resulting in increased PAE risk during interventions. Further research should be done to verify the hypothesis and the mechanism. Another interesting finding was that hypertension was an independent protective predictor of primary patency. We noticed that there were no similar reports in the literatures. The mechanism need to be studied in the future.

The treatments of PAE include aspiration, local thrombolysis, angioplasty with balloons and embolectomy. Keeping in mind that PAE is an acute limb ischemia that could lead to limb loss, and prompt restoration of the popliteal flow is urged. Angioplasty with balloons should be carried out in time if aspiration or local thrombolysis were not working. The possible mechanism of balloon angioplasty is that it may help those emboli made of soft atheromatous plaque to break into tiny particles and be flushed into distal vessels, or crush the emboli into a distal vessel from the popliteal artery. If the endovascular techniques do not work, embolectomy under general anesthesia should be carried out to open popliteal artery without delay. At least one runoff vessel flow should be restored before the procedure can be terminated after a PAE event. Our experience suggests that PAE is typically reversible by comprehensive techniques.

The concern for DE has led to a debate on the use of EPDs during lower extremity intervention. Published reports have evaluated the use of several kinds of EPDs and confirmed of the presence of debris [6], [11]. However, since these studies did not involve any control groups to confirm the short term or long term clinical efficacy of EPDs, so far it can only demonstrate that certain EPDs are safe and efficient in collecting debris during the procedure. In the Preventing Lower Extremity Distal Embolization Using Embolic Filter Protection (PROTECT) study recruiting 56 lesions treated in 40 patients all with EPDs, a side branch embolization occurred in one patient, and a no-flow phenomenon occurred in another as a result of an overfilled filter [3]. The potential benefit of EPDs must therefore be balanced against the associated complications. A study by Shrikhande and colleagues indicates that DE is typically reversible with endovascular techniques and is not associated with unfavorable clinical outcomes in a 24-month follow up, suggesting that EPDs may be unnecessary for lower extremity intervention [2]. On the other hand, the use of distal protection to prevent DE during intervention in carotid angioplasty and stenting is well established [16], [17], [18]. It is reasonable to believe that EPDs might be beneficial for some certain lesion types and treatment methods in lower extremity interventions. This study focus on chronic SFA arteriosclerosis cases underwent primary PTA + stent. Our data suggests that PAE events are not associated with significant lower long-term primary patency, secondary patency or limb salvage rates in both Kaplan-Meier analysis and Cox multivariate regression, if the popliteal flow is restored in time. This is similar to some previous research that complete resolution of macroembolization may not affect long-term patency and limb salvage [2]. The results do not support routine application of EPDs. However, these results must be viewed with caution.

This study has some limitations that require attention. First, we only focus on chronic SFA arteriosclerosis cases underwent primary PTA + stent, other lesion types treated by other methods are not included in this study. Reinterventions and some new atherectomy devices are associated with higher DE rate [19], [20], thus more likely to benefit from EPDs, but not discussed here. Second, the PAE rate is so low that the sheer event number is small. Thus the power to detect differences in outcome may be insufficient. Third, patency rates and limb salvage can only present a part of the clinical outcome. Distal neuromuscular function was not compared, the assessment of other collateral runoff vessels in the foot was not routinely performed neither. In addition, the mean age was less than 70 years old, which means this is a young cohort for arteriosclerosis patients who can somehow compensate from the ischemia. Therefore we don't know if EPDs could improve long-term distal neuromuscular function in patients with more advanced age.

## Conclusions

PAE is a rare event that occurs in primary SFA PTA + stent. TASC C/D lesion, CTO and history of cerebral ischemic stroke are risk factors for PAE. PAE is typically reversible by comprehensive techniques. If the popliteal flow is restored in time, PAE has no significant effect on SFA patency and limb salvage rates.

## References

[1] Davies MG, Bismuth J, Saad WE, Naoum JJ, Mohiuddin IT, et al (2010) Implications of in situ thrombosis and distal embolization during superficial femoral artery endoluminal intervention. Ann Vasc Surg 24: 14–22. 19748214 10.1016/j.avsg.2009.06.020

[2] Shrikhande GV, Khan SZ, Hussain HG, Dayal R, McKinsey JF, et al (2011) Lesion types and device characteristics that predict distal embolization during percutaneous lower extremity interventions. J Vasc Surg 53: 347–352. 21129906 10.1016/j.jvs.2010.09.008

[3] Shammas NW, Dippel EJ, Coiner D, Shammas GA, Jerin M, et al (2008) Preventing lower extremity distal embolization using embolic filter protection: results of the PROTECT registry. J Endovasc Ther 15: 270–276. 18540705 10.1583/08-2397.1

[4] Muller-Hulsbeck S, Schafer PJ, Humme TH, Charalambous N, Elhoft H, et al (2009) Embolic protection devices for peripheral application: wasteful or useful? J Endovasc Ther 16 Suppl 1 I163–I169. 19317576 10.1583/08-2596.1

[5] Zeller T, Schmidt A, Rastan A, Noory E, Sixt S, et al (2012) Initial Experience With the 5×300-mm Proteus Embolic Capture Angioplasty Balloon in the Treatment of Peripheral Vascular Disease. J Endovasc Ther 19: 826–833. 23210883 10.1583/JEVT-12-3960MR.1

[6] Hadidi OF, Mohammad A, Zankar A, Brilakis ES, Banerjee S (2012) Embolic capture angioplasty in peripheral artery interventions. J Endovasc Ther 19: 611–616. 23046325 10.1583/JEVT-12-3977MR.1

[7] Karnabatidis D, Katsanos K, Kagadis GC, Ravazoula P, Diamantopoulos A, et al (2006) Distal embolism during percutaneous revascularization of infra-aortic arterial occlusive disease: an underestimated phenomenon. J Endovasc Ther 13: 269–280. 16784313 10.1583/05-1771.1

[8] Lam RC, Shah S, Faries PL, McKinsey JF, Kent KC, et al (2007) Incidence and clinical significance of distal embolization during percutaneous interventions involving the superficial femoral artery. J Vasc Surg 46: 1155–1159. 18154991 10.1016/j.jvs.2007.07.058

[9] Lookstein RA, Lewis S (2010) Distal embolic protection for infrainguinal interventions: how to and when? Tech Vasc Interv Radiol 13: 54–58. 20123433 10.1053/j.tvir.2009.10.007

[10] Norgren L, Hiatt WR, Dormandy JA, Nehler MR, Harris KA, et al. (2007) Inter-Society Consensus for the Management of Peripheral Arterial Disease (TASC II). J Vasc Surg 45 Suppl S: S5–S67. 10.1016/j.jvs.2006.12.037 17223489

[11] Shammas NW, Coiner D, Shammas GA, Christensen L, Dippel EJ, et al (2009) Distal embolic event protection using excimer laser ablation in peripheral vascular interventions: results of the DEEP EMBOLI registry. J Endovasc Ther 16: 197–202. 19456199 10.1583/08-2642.1

[12] Banerjee S, Iqbal A, Sun S, Master R, Brilakis ES (2011) Peripheral embolic events during endovascular treatment of infra-inguinal chronic total occlusion. Cardiovasc Revasc Med 12: 134–137. 10.1016/j.carrev.2010.10.005 21195034

[13] Matsi PJ, Manninen HI (1998) Complications of lower-limb percutaneous transluminal angioplasty: a prospective analysis of 410 procedures on 295 consecutive patients. Cardiovasc Intervent Radiol 21: 361–366. 9853140 10.1007/s002709900281

[14] Schlittenhardt D, Schober A, Strelau J, Bonaterra GA, Schmiedt W, et al (2004) Involvement of growth differentiation factor-15/macrophage inhibitory cytokine-1 (GDF-15/MIC-1) in oxLDL-induced apoptosis of human macrophages in vitro and in arteriosclerotic lesions. Cell Tissue Res 318: 325–333. 15459768 10.1007/s00441-004-0986-3

[15] Fatini C, Sofi F, Gensini F, Sticchi E, Lari B, et al (2004) Influence of eNOS gene polymorphisms on carotid atherosclerosis. Eur J Vasc Endovasc Surg 27: 540–544. 15079780 10.1016/j.ejvs.2004.02.008

[16] Bosiers M, Deloose K, Verbist J, Peeters P (2008) The impact of embolic protection device and stent design on the outcome of CAS. Perspect Vasc Surg Endovasc Ther 20: 272–279. 18930936 10.1177/1531003508323730

[17] Goodney PP, Schermerhorn ML, Powell RJ (2006) Current status of carotid artery stenting. J Vasc Surg 43: 406–411. 16476626 10.1016/j.jvs.2005.11.012

[18] Cohen SN (2007) Incidence of new brain lesions after carotid stenting with and without cerebral protection. Stroke 38: e18, e19–e20. 10.1161/STROKEAHA.106.478164 17363717

[19] Shammas NW, Coiner D, Shammas GA, Dippel EJ, Christensen L, et al (2011) Percutaneous lower-extremity arterial interventions with primary balloon angioplasty versus Silverhawk atherectomy and adjunctive balloon angioplasty: randomized trial. J Vasc Interv Radiol 22: 1223–1228. 21757372 10.1016/j.jvir.2011.05.013

[20] Suri R, Wholey MH, Postoak D, Hagino RT, Toursarkissian B (2006) Distal embolic protection during femoropopliteal atherectomy. Catheter Cardiovasc Interv 67: 417–422. 16489560 10.1002/ccd.20634

